# Association of catechol-o-methyltransferase gene polymorphism with preeclampsia and biomarkers of oxidative stress: Study protocol for a prospective case-control study in Pakistan

**DOI:** 10.1371/journal.pone.0304314

**Published:** 2024-06-11

**Authors:** Farheen Yousuf, Tasneem Fatima, Rehana Rehman, Iqbal Azam, Samra Khan, Maha Anis, Rubeka Mansha, Shagufta Khan

**Affiliations:** 1 Department of Obstetrics & Gynaecology, The Aga Khan University, Karachi, Pakistan; 2 Department of Anatomy, Bahria University Medical and Dental College, Karachi, Pakistan; 3 Department of Biological and Biomedical Sciences, The Aga Khan University, Karachi, Pakistan; 4 Department of Community Health Sciences, The Aga Khan University, Karachi, Pakistan; University of Dhaka, BANGLADESH

## Abstract

**Background:**

Preeclampsia is one of the three leading causes of worldwide maternal mortality. Oxidative stress-mediated endothelial damage is expected to be an ultimate common mechanism in the pathophysiology of preeclampsia. The role of bioamines is also well-established in the induction of preeclampsia. This project is aimed to understand the factors which may affect the induction, progression, and aggravation of preeclampsia and oxidative stress during pregnancy. This study will explore the methylation pattern of the Catechol-O-methyltransferase gene to determine its role in the pathogenesis of preeclampsia, association of Val158Met polymorphism with a wide range of oxidative stress biomarkers, major antioxidants vitamins, and blood pressure regulating amines in preeclamptic Pakistani women.

**Methods and analysis:**

In this prospective case-control study, 85 preeclamptic and 85 normotensive pregnant women will be recruited in their third trimesters. DNA will be extracted from peripheral blood and Val158Met polymorphism in the Catechol-O-methyltransferase gene will be examined on PCR amplified product digested with Hin1II (NlaIII) restriction enzyme, further validated by Sanger sequencing. Methylation-sensitive PCR will also be performed. Oxidative stress biomarkers, antioxidant vitamins, bioamines, and catechol-O-methyltransferase levels will be measured by ELISA. The data will be used to correlate maternal and fetal outcomes in both groups.

**Discussion:**

This study will help to identify and understand the multifactorial path and cause-effect relationship of gene polymorphism, oxidative stress biomarkers, major antioxidants vitamins, and blood pressure regulating amines in the pathogenesis and aggravation of preeclampsia in the Pakistani population. The outcome of this project will be particularly helpful in reducing the incidence of preeclampsia and further improving its management.

## Introduction

Normal pregnancy is accompanied by several physiological and metabolic changes that result in high metabolic demand [1]. This increased demand for tissue oxygen leads to increased oxidative stress and antioxidant defense mechanisms [2]. Oxidative stress (OS) affects multiple physiological processes and is also involved in the onset of pre-term labor [3]. Hypertension is one of the most prevalent issues during pregnancy, complicating 5–10% of pregnancies [4]. Several diseases have been known to have an underlying inflammatory component with altered redox balance [5]. Hypertension is one of the vascular disorders that arise due to high oxidant concentration [6]. There is sufficient evidence that both endothelial dysfunction and inflammation cascade lead to inflammation which ultimately causes the development of hypertension [7].

Preeclampsia (PE), a hypertensive pregnancy disorder, is one of the three leading causes of maternal mortality worldwide [8]. Oxidative stress-mediated endothelial damage may be the ultimate common mechanism causing tissue damage and is crucial in the pathophysiology of PE [9]. PE etiology is complicated, involving both genetic and environmental factors. These genetic factors are thought to be contributing to almost 50% of PE cases [10].

Catechol-O-methyltransferase (COMT) is a gene that codes for the enzyme catechol-O-methyltransferase, which oversees most of the catecholamine degradation [11]. The dopaminergic pathway plays an important role in blood pressure regulation [12]. Catechol drugs used to treat hypertension are also one of the substrates of the COMT enzyme [13]. Three common single-nucleotide polymorphisms (SNP) have been reported for the COMT gene. However, Val158Met, where nucleotide guanine is replaced by adenine at 158^th^ position on exon 4, is more often associated with different disease conditions due to reduced activity of COMT [14]. Several studies have shown that the Val158Met polymorphism is involved in the development of PE in many populations, such as pregnant Korean and Norwegian women [15–18].

Given the global morbidity and mortality caused by PE, particularly its impact on maternal health in Pakistani women, the number of previous PE studies is scarce. Furthermore, these studies cover a variety of topics, such as community perceptions of PE, epidemiology, risk factors, and associations with various PE parameters [19–21]. More research is needed to uncover the unknown aspects of PE pathophysiology at the molecular level and its relationship with OS in Pakistani women. To our knowledge, no study has been published that links the COMT gene Val158Met polymorphism to PE and OS biomarkers in the Pakistani population. Our hypothesis is that preeclampsia is associated with the Val158Met COMT gene polymorphism along with the disruption of oxidative stress in Pakistani pregnant women. This study will play an important role in identifying and developing a biomarker; as well as understanding the factors that may affect the progression and aggravation of PE and OS during pregnancy. The outcomes of this study will also define the factors that are associated with OS; therefore, taking measures to keep the OS under acceptable levels during pregnancy. Thus, our proposed study is designed.

## Aims & objectives

This study is designed to:

- Explore the association of COMT gene Val158Met polymorphism with the progression of PE in the Pakistani population.
- Evaluate the correlation COMT gene Val158Met polymorphism with bioamines which contributes to the pathogenesis of PE in Pakistani women.
- Assess the role of COMT gene Val158Met polymorphism with biomarkers of oxidative stress in normotensive and PE pregnant women.
- Correlate maternal and fetal outcomes COMT gene Val158Met polymorphism with biomarkers of oxidative stress in normotensive and PE pregnant women.

## Materials and methods

### Study design

**Fig 1:**
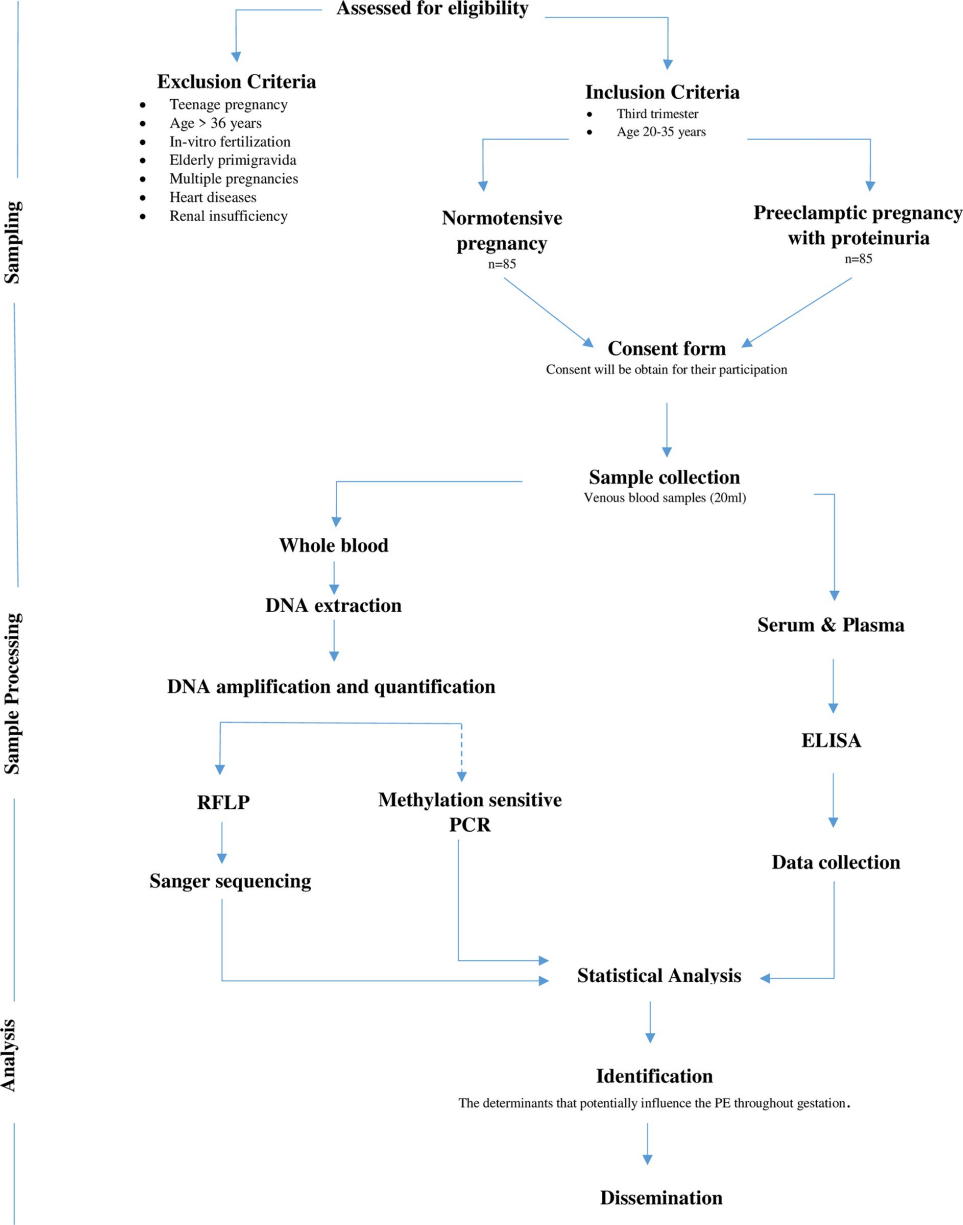
Schematic figure of study design.

In this prospective case-control study, eligible study participants i.e. normotensive and preeclamptic pregnant women in third trimesters, will be recruited after obtaining patient’s informed written or thumb expression consent in the presence of a witness and their rights will be protected as per the guiding principles of the Helsinki Declaration. Eligible pregnant women in their third trimesters will be recruited from the labor room, special care of B2 ward, and Obs & Gynae words (A2 and B2 wards) at The Aga Khan University Hospital (AKUH), Karachi, Pakistan. An ERC-approved questionnaire including complete medical and gynecological history will be filled out with the help of participants. The sampling for this study started on 15 November 2022 and the timeline of this study is three years. This study is approved by the Ethics Review Committee, The Aga Khan University, Pakistan (Ref# 2021-1911-18538). Fig 1 outlines the study design.

### Sample size

We used Open Epi calculator to estimate sample size [22]. The prevalence of PE was kept at 10% as reported by WHO with 5% CI [23]. a total of 120 participants; 60 preeclampsia patients and 60 healthy pregnant will achieve 80% power with an exposure of expected prevalence of COMT gene polymorphism ranging between 10–20% with an absolute increase of at least 25% in preeclamptic women and a level of 5% significance. However, we increased our total sample size to 170 (85 each) to achieve more statistical power.

### Eligibility criteria

An experienced gynaecologist will monitor the patients admitted to the labor room and filter them as per the study’s inclusion and exclusion criteria. Pregnant women in third trimesters (20–35 years) diagnosed with PE and normotensive pregnant women in third trimesters (20–35 years) will be included in this study as test and control participants, respectively. Women will be characterized as having PE as per the definition given by the American College of Obstetricians and Gynaecologists (ACOG). Furthermore, normotensive patients will be defined as pregnant women with a lack of hypertension and proteinuria.

Teenage pregnancy, women above 36 years of age, women undergoing in-vitro fertilization, elderly primigravida, and multiple pregnancies will not be included in this study. Tobacco adductors, illnesses like severe anemia, gestational or otherwise diabetes mellitus, cardiac condition, essential hypertension, renal insufficiency, history of stroke, and alcoholics will be excluded. Women with cardiac disease and jaundice will also be omitted.

### Sample collection

Venous blood samples (20 ml) of both cases and control pregnant women will be taken by doctor or trained staff personnel in hospital setting ensuring maximum patient safety and following standard hospital guidelines. 10 ml of each blood sample will be collected into an EDTA tube and the remaining 10 ml of blood samples collected into a Gel tube to generate plasma and serum, respectively. Both samples will be stored in aliquots at ˗80°C until further processing.

### Sample processing

The Genomic DNA purification kit (Thermo Scientific, USA) kit will be used to extract DNA from blood samples. The quantity and quality of the genomic DNA will be checked on a NanoDrop spectrophotometer and 1% gel with Gene Ruler 1 kb DNA Ladder (Thermo Scientific, USA), stained with Ethidium bromide respectively. PCR will be performed using a modified method. Briefly, a total reaction mixture of 25 μl will be used having 500ng DNA, 0.2 mM dNTPs, 2.5 mM MgCl2, 0.05 U Taq, 10% DMSO, 1 μl Taq buffer, and 0.5 μM primers of COMT using the sequences [24] 5´-ACTGTGGCTACTCAGCTGTG-3´ (forward) and 5´-CCTTTTTCCAGGTCTGACAA-3´ (reverse). 13.8 μl deionized water will be used to make up the volume. The thermal cycler (Eppendorf, Denmark) will be set at 94°C during the denaturation step, 66°C during the annealing step, and 72°C during the final extension step. The amplicons will be resolved using 1.5–2% agarose gel with ethidium bromide and visualized under UV with a 50-base pair ladder. (Thermo Scientific, USA). Moreover, methylation-sensitive PCR will be run to check the methylation status of the COMT gene using EpiTect MSP Kit (QIAGEN Hilden, Germany).

To visualize the presence of Val158Met polymorphism, the obtained PCR amplified product will be digested with Hin1II (NlaIII) restriction enzyme (Thermo Scientific, USA) [24].

The presence of adenine at the specified codon will yield four fragments of 96, 26, 29, and 18 bp by the digestion of 169bp amplicon. On the contrary, the presence of guanine at the site will yield three fragments of 114, 26, and 29bp due to the lack of NlaIII recognition site.

Selected positive samples for COMT Val158Met mutation will be sent to Eurofins for Sanger sequencing.

### Estimation of bioamine level & oxidative stress biomarkers

Blood pressure regulating amines such as dopamine and its transporter, serotonin, adrenaline, and noradrenaline levels, and oxidative stress biomarkers including glutathione peroxidase, glutathione reductase, superoxide dismutase, malondialdehyde, catalases, total oxidant status, and DNA damage (Human 8-Hydroxy-deoxyguanosine) will be measured in samples through ELISA kits’ method by BT-Labs. Similarly, the major antioxidants vitamins including vitamins A, C, E, and B9 levels will be measured in the samples through the ELISA kits’ method by BT-Labs. These ELISA tests will be performed using an ELISA plate reader (BIO-RAD, USA).

### Measurement of catechol-o-methyl transferase levels

Levels of catechol-o-methyl transferase enzyme levels will be measured using the ELISA kit method by BT-Lab using an ELISA plate reader (BIO-RAD, USA).

### Correlation of maternal and fetal outcomes

Maternal and fetal outcomes such as blood pressure, family history of BP, maternal age, and comorbidities as well as mode of delivery, fetal birth weight, intrauterine growth restriction, respiratory distress, and stillbirth will be correlated with COMT gene Val158Met polymorphism with biomarkers of oxidative stress in normotensive and PE pregnant women using maternal outcomes proforma.

### Data acquisition and management

The related proforma will be completed by an experienced member of the project team. At DBBS, Aga Khan University, an electronic data management system (DMS) will be built, and all data will be archived in Word and Excel sheets. For this study, each participant will be assigned a code to protect their identity. The data will be archived for no more than 5 years. Access to the hard copies and identities will be restricted to the PI only.

### Statistical analysis

The data will be examined using IBM-SPSS version 19.0 statistical software. The results will be presented as the mean SEM (standard error of the mean). To determine Hardy-Weinberg equilibrium in cases and controls, the chi-square test will be utilized. To examine genotype distribution and allele frequencies between groups, the chi-square test will be performed. An odds ratio with a 95% confidence interval was used to assess the relative risk of preeclampsia associated with the COMT Val158Met polymorphism. To compare variable levels between genotype groups, analysis of variance was utilized (ANOVA). Statistical significance is defined as a p-value of 0.05 or less. Each ELISA test will be carried out three times.

### Patient and public involvement

No patient or public is involved.

## Discussion

Due to unclear etiology, preeclampsia remains a severe burden in the developing world [25]. Although sometimes preeclampsia follows a Mendelian pattern of disease for a small number of populations, mostly the disorder is a result of complex genetic anomalies. A wide range of case-control studies has been published employing a candidate gene approach for preeclampsia. This approach includes the selection of a single gene, that was identified in linkage studies, and then investigating the frequency of its allelic variants [26]. Furthermore, we will also assess the methylation pattern of the COMT gene in PE women. This will allow us to determine the altered DNA methylation pattern in patients which may lead to PE pathogenesis. Herein we will investigate the association of the COMT gene specifically its Val158Met dominant variant in the pathophysiology of PE in the Pakistani population. Another aspect of our study would be to link this variant with oxidative stress biomarkers so that a multifactorial path could be investigated concerning our local population. The findings of the investigation will be disseminated among the scientific circle through presentations (oral and poster) and publications in peer-reviewed journals.

## Strengths and limitations

This study will employ candidate gene linkage with a holistic approach i.e., associating the mutation with a wide range of oxidative stress biomarkers, major antioxidants vitamins, and blood pressure regulating amines in preeclamptic Pakistani women and also will assess the methylation pattern of the Catechol-O-methyl transferase gene to determine the role of altered methylation status in the pathogenesis of preeclampsia. Sufficient statistical power will help to analyze the frequency of the variant in the Pakistani population. Validation of Val158Met SNP will be achieved by Sanger Sequencing.

All the participants will be enrolled from a single hospital thus it could serve as one of the limitations of the study. Moreover, we will not be measuring biomarkers at multiple time points during the pregnancy thereby limiting the potential for enhanced understanding of the oxidative stress dynamics in preeclampsia. As a result, the capacity to generalize findings to the larger pregnant population may be limited.
